# Bursted BMP Triggered Receptor Kinase Activity Drives Smad1 Mediated Long-Term Target Gene Oscillation in c2c12 Cells

**DOI:** 10.1371/journal.pone.0059442

**Published:** 2013-04-01

**Authors:** Daniela Schul, Alexandra Schmitt, Janine Regneri, Manfred Schartl, Toni Ulrich Wagner

**Affiliations:** Physiological Chemistry I, University of Wuerzburg, Wuerzburg, Germany; Temple University, United States of America

## Abstract

Bone Morphogenetic Proteins (BMPs) are important growth factors that regulate many cellular processes. During embryogenesis they act as morphogens and play a critical role during organ development. They influence cell fates via concentration-gradients in the embryos where cells transduce this extracellular information into gene expression profiles and cell fate decisions. How receiving cells decode and quantify BMP2/4 signals is hardly understood. There is little data on the quantitative relationships between signal input, transducing molecules, their states and location, and ultimately their ability to integrate graded systemic inputs and generate qualitative responses. Understanding this signaling network on a quantitative level should be considered a prerequisite for efficient pathway modulation, as the BMP pathway is a prime target for therapeutic invention. Hence, we quantified the spatial distribution of the main signal transducer of the BMP2/4 pathway in response to different types and levels of stimuli in c2c12 cells. We found that the subcellular localization of Smad1 is independent of ligand concentration. In contrast, Smad1 phosphorylation levels relate proportionally to BMP2 ligand concentrations and they are entirely located in the nucleus. Interestingly, we found that BMP2 stimulates target gene expression in non-linear, wave-like forms. Amplitudes showed a clear concentration-dependency, for sustained and transient stimulation. We found that even burst-stimulation triggers gene-expression wave-like modulations that are detectable for at least 30 h. Finally, we show here that target gene expression oscillations depend on receptor kinase activity, as the kinase drives further expression pulses without receptor reactivation and the target gene expression breaks off after inhibitor treatment in c2c12 cells.

## Introduction

Tightly controlled quantitative integration of ligand levels is very important for multicellular organisms. This is best illustrated by embryonic development. Bone Morphogenetic Proteins (BMPs) act as graded morphogens and are responsible for the dorsal-to-ventral cell type specification in a dose-dependent manner [1]. Clearly, there must exist a machinery that translates these morphogen gradients into so far unknown cell fate mechanisms by thresholding them.

BMPs are secreted proteins that belong to the TGF-ß superfamily. They are involved in the regulation of many cellular processes like proliferation, differentiation, adhesion as well as apoptosis [2]–[4]. The ligands signal through a family of transmembrane serine/threonine kinase receptors. Signaling occurs through the heterotetramerization of two receptor subtypes [5]. The constitutively active type II receptor activates the type I receptor by trans-phosphorylation, and the activated type I receptor then in turn activates the key signal transducers, the Smad proteins [6]. Distinct Smad family members have been identified and classified into three different subgroups. The receptor-regulated Smads (R-Smads) include Smad1, 2, 3, 5 and 8. They become phosphorylated by the type I receptor kinases. Smad2 and Smad3 are activated by TGF-ß proteins, while Smad1, 5 and 8 are activated by BMP ligands. The R-Smads form complexes with Smad4, which is the only member of the common mediator Smads (Co-Smads) [7]. The Smad complexes translocate into the nucleus and activate expression of target genes in association with other transcription factors [8]. However, even without ligand the cellular distribution of Smad2 and Smad3 is not static, but the proteins are constantly shuttling between the nucleus and the cytoplasm [9]. In the case of BMP signaling, Smad-complexes bind to short GC-rich DNA regions in target gene promoters that were identified as BMP-responsive elements (BRE) [10]. The inhibitory Smad proteins, Smad6 and Smad7, stably bind to the intracellular domain of the type I receptor and thereby prevent the phosphorylation of R-Smads [11], [12]. Smad6 also exhibits other inhibitory functions like competing with Smad4 for binding to phosphorylated Smad1 [13] and interacting with transcriptional co-repressors in the nucleus [14].

The BMP signaling pathway is implicated in severe human diseases like cancer, fibrosis, multiple hereditary conditions and wound-healing disorders [15]–[18]. This elucidates that its regulation needs to be tightly controlled and is often subject to disregulation. Consequently, it is reasonable to expect a strict fine tuning of each individual step of signal transduction when looking at the large number of modulatory factors that regulate the pathway (reviewed in [19]). In order to successfully develop new therapeutics, it is thus pivotal to quantify the BMP signal transduction dynamics, their modulation and coupled transcriptional outcomes with high resolution and accuracy over an extended period of time. In this context a large number of studies have been published on the TGFß-Smad2/3 pathway. First, indirect immunostaining had shown that Smad2 and Smad3 almost completely translocate into the nucleus after 30 minutes of stimulation with TGF-ß [20]. Later, the kinetics of the Smad2 nucleocytoplasmic shuttling have been investigated with and without stimulation using GFP-fusions. It revealed, that the nuclear export rate for non-induced cells is more rapid than the import rate and that the nuclear accumulation of Smad2 upon stimulation is caused by a pronounced drop in the export rate [9]. Recently, a study on quantification of TGF-ß signaling showed that both dose as well as time course of stimulation have significant effects on Smad2 signaling dynamics. Interestingly, this study also demonstrated that cells do respond to short time stimulation pulses [21].

In principle, there are several ways to modulate signaling output. Besides ligand concentration, stimulation time obviously is an important signal modulator. Studies on ERK revealed that duration of its activation controls cell fate by proliferative or anti-proliferative responses [22], [23]. Similar results were obtained for the TGFß-Smad2/3 pathway, where variation in the cellular behavior was evoked by different signaling durations [24].

Investigating the quantitative integration of BMP signaling, we present long-term measurements of cellular responses to both sustained and transient BMP2 stimulation under varying concentrations in c2c12 cells. Spatio-temporal analyses of Smad1 distribution revealed a basal shuttling mechanism that is unaffected by ligand stimulation. Moreover, the transcriptional responses were evaluated by two independent approaches. The gene expression induced by BMP results in oscillating curve patterns over time. Mathematical Fourier analysis of our measurements revealed two oscillation components that clearly responded to modulation of stimulation parameters and also verified that basal oscillation is fully dependent on receptor kinase activity in c2c12 cells.

## Results

### Smad1 shuttles constantly between nucleus and cytoplasm while the amount of pSmad increases in relation to the stimulation concentration

**Figure 1:**
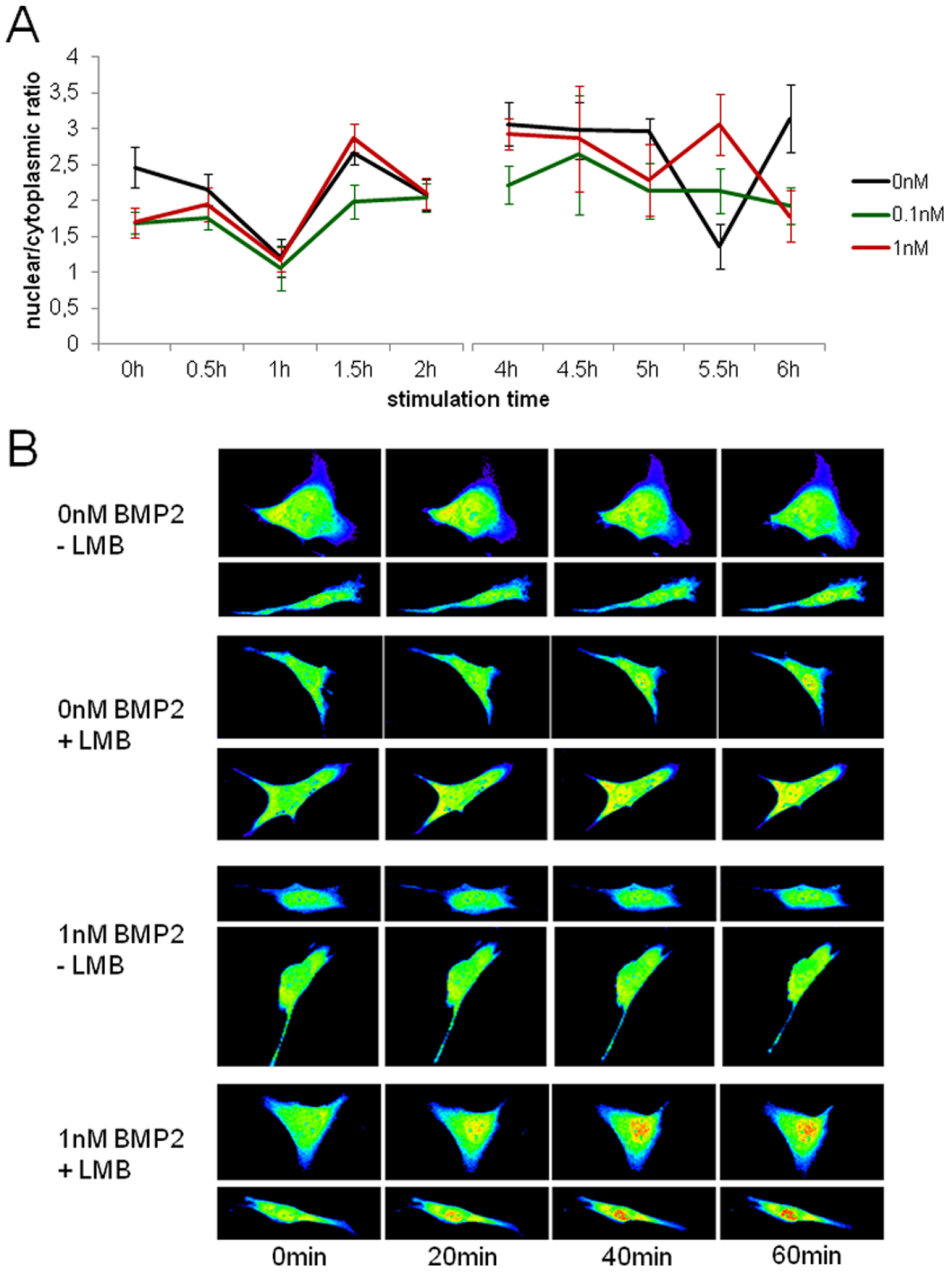
Nucleo/cytoplasmic shuttling of Smad1. (A) 3D-Analysis of immunofluorescent stainings using an anti-Smad1 primary antibody, secondary Alexa488 antibody, specific membrane- and nuclear staining. Confocal stacks were processed using the Volocity 3D image analysis software; the nuclear/cytoplasmic ratio was calculated and illustrated. The black line depicts the time course of the ratio for the unstimulated control, the green line shows the subcellular distribution after stimulation with 0.1 nM BMP2 and the red line after 1 nM BMP2 stimulation. The depicted values are the average of eleven cells for every time point and concentration. (B) Analysis of the Smad1-live shuttling using a GFP-Smad1 fusion protein. Cells were transiently cotransfected with the Smad1-GFP fusion construct and a H2B-mCherry vector and starved over night. On the next day, cells were stimulated with either 0 nM BMP2 or 1 nM BMP alone or while adding Leptomycin B and imaged at the indicated time points after stimulation using a confocal microscope. The figure shows two independent cells for each treatment over time. The fluorescence intensity of Smad1-GFP is indicated in pseudocolors ranging from dark blue (lowest signal) to red (strongest signal).

In order to measure the subcellular localization of Smad1 under non-stimulated and stimulated conditions, we performed indirect immunofluorescence staining using anti-Smad1 in c2c12 cells. For quantification of Smad1 amounts in the nucleus and the cytoplasm, respectively, we further stained cell membranes and nuclei with specific dyes. Confocal analysis revealed, that there is no significant difference in Smad1 subcellular localization upon ligand stimulation with 0.1 nM or 1 nM BMP2 over 2 h (Fig. 1A; Figure S1). Instead both, the non-stimulated as well as the stimulated cells, showed a nucleocytoplasmic oscillatory translocation behavior of Smad1.

We next used a GFP-Smad1 fusion construct and transiently cotransfected this with a H2B-mCherry vector into c2c12 wildtype cells to verify this finding. Transfected cells were either non-stimulated or stimulated with 1 nM BMP2, but again did not find nuclear accumulation of the GFP-Smad1 fusion protein in either treatment up to 1 h after stimulation start. To ensure that the fusion protein was able to visualize nuclear accumulation of Smad1, we added the nuclear-export-inhibitor Leptomycin B (LMB) to the previously treated cells. Under this condition, GFP-Smad1 fusion proteins clearly accumulated in the nuclei of LMB treated cells independent of previous BMP stimulation (Fig. 1B; Figure S2). The quite different cell morphology is typical for this cell type (Figure S4). These findings indicate that Smad1 shuttles constantly between the nucleus and the cytoplasm. Furthermore, the distribution of Smad1 protein due to this basal nucleocytoplasmic shuttling is not significantly altered by stimulation with either 0.1 nM or 1 nM BMP2. However, we found that the amount of nuclear phospho-Smad1 increases in a BMP2 stimulation time- and concentration-dependent manner (Figure S3A–D).

### Sustained and oscillating gene expression upon continuous stimulation with BMP2

**Figure 2:**
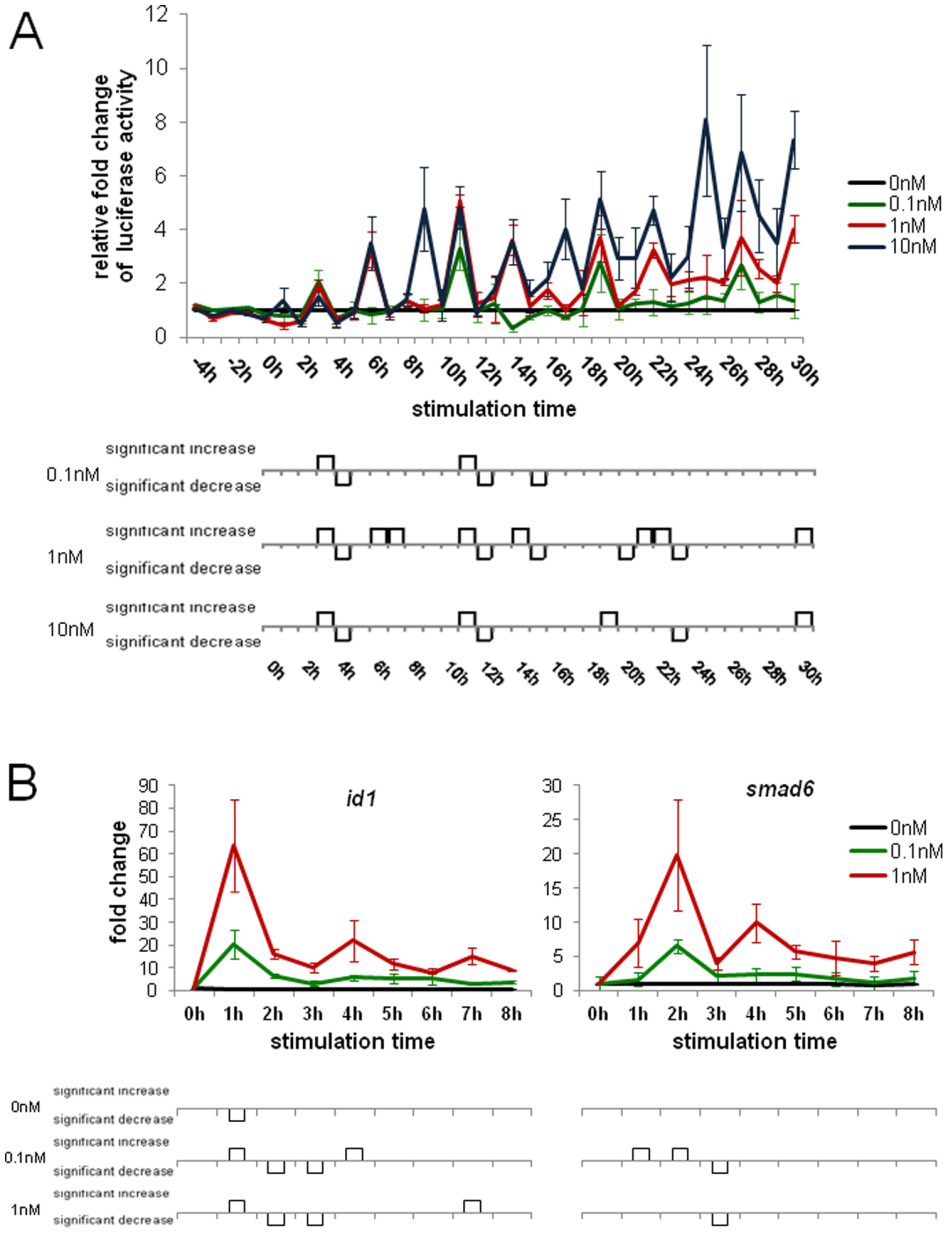
Gene expression analysis of continuously stimulated cells. (A) 30 h experiment using cells that stably express the Gaussia Luciferase under control of a BRE minimal promoter. The cells were stimulated with either 0.1 nM (green), 1 nM (red) or 10 nM (blue) BMP2 or non-stimulated (black) as control, 50 µl medium were removed every hour and the Luciferase activity was measured. The relative fold change to the unstimulated control was calculated and assigned. (B) Quantitative real-time PCR was performed on the BMP target genes *id1* and *smad6.* The cells were stimulated with 0.1 nM (green), 1 nM (red) BMP2 or non-stimulated (black) and every hour one sample was lysed and frozen at −80°C until the further processing.

We next investigated the time course of target gene expression of cells continuously stimulated with BMP2. We therefore generated a clonal cell line stably expressing the secreted Gaussia Luciferase under control of a BRE minimal promoter. Using secreted Luciferase allowed us to record a true time course of BMP induced promoter transcriptional regulation, as only a small volume of supernatant was taken from each cell culture well at each time point. We analyzed Luciferase activity over a time course of 4 h before to 30 h after stimulation. Total Luciferase activity was concentration dependent, which is clear evidence for a functional read-out system as well as the lag-phase at the beginning (Figure S5). Furthermore, as expected, Luciferase activity decreased to baseline levels when incubated at 37°C for 1 h (Figure S6, [25]). We found that BMP-induced reporter gene expression is concentration- and time-dependent (figure 2A), and oscillating progressions suggest gene expression pulses during continuous stimulation with BMP2. Interestingly, the frequencies of the activity bursts are similar for all tested ligand concentrations, however, the wave-peak amplitudes show clear positive correlation with the respective BMP2-concentration used for stimulation (Fig. 2A, Figure S8A and B). To further prove the reliability of the reporter system, we performed transient experiments with a NFkb-binding site upon stimulation with TNFα (Figure S7).

In order to verify this finding in an independent experimental setup, we performed qRT-PCR on the well-described BMP target genes *id1* and *smad6*. Both genes showed an oscillating expression profile with a mRNA maximum after 1 h or 2 h, for 0.1 nM and 1 nM BMP2 respectively (Fig. 2B; Figure S9, Figure S10 and Figure S11). At later time points, the mRNA expression pulses decreased gradually to a low level of oscillations that were detectable until 30 h after stimulation (Figure S12). We could show that these transcript level oscillations do directly reflect changes of the target gene mRNA levels, but not changes of the housekeeping gene levels used for RNA level calibrations (Figure S13A and B).

However, the results from these experiments differ in the oscillatory pattern as the Luciferase activity increases over time whereas the mRNA levels decrease. This could be attributed to many reasons including different translation dynamics, different promoters (optimized BRE-promoter element in contrast to endogenous promoters), measurement of protein activity in contrast to mRNA level, half-life times of mRNAs and proteins as well as the delay of the protein due to export, synthesis and folding. But intracellular Gaussia Luciferase mRNA levels show a similar pattern to endogenous target genes (Figure S14).

### Short-time receptor stimulation shows similar gene expression profile as continuous stimulation

**Figure 3:**
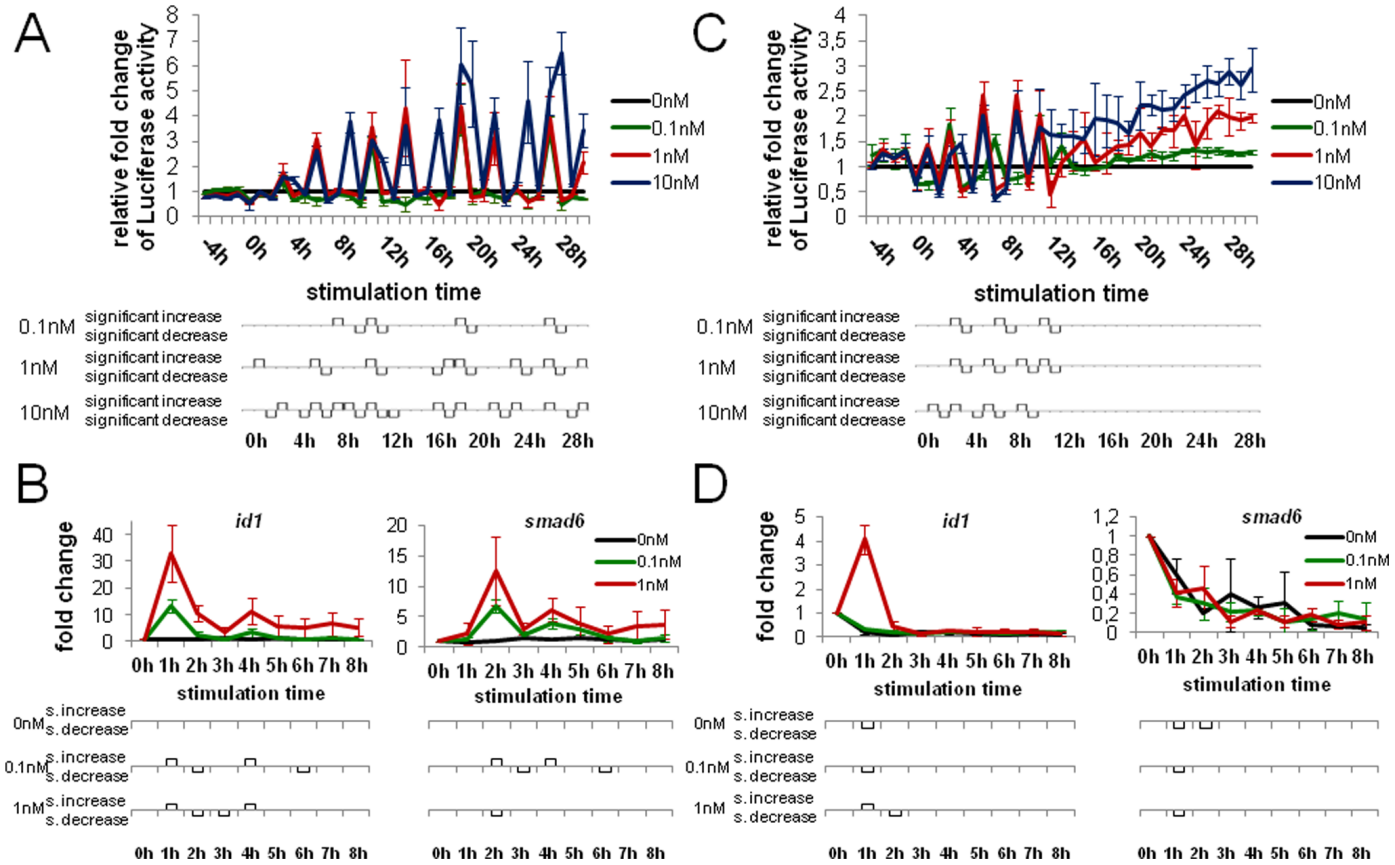
Gene expression analysis of short-time receptor stimulus and short-time Smad-phosphorylation. (A) 30 h experiment using the stable reporter cell line. The cells were stimulated with 0.1 nM (green), 1 nM (red) 10 nM (blue) BMP2 or non-stimulated (black) and after 15 minutes the stimulation medium was removed and fresh starvation medium were given to the cells. Then every hour 50 µl medium was withdrawn and the Luciferase activity was measured. The relative fold change to the unstimulated control is depicted. (B) Quantitative real-time PCR on *id1* and *smad6.* The cells were stimulated with 0.1 nM (green), 1 nM (red) BMP2 or non-stimulated (black) for 15 minutes and then cultivated in starvation medium until cell lysis. (C) 30 h experiment using the stable reporter cell line. The cells were stimulated with the indicated ligand concentrations and after 15 minutes Dorsomorphin was added to the cells. 50 µl medium were withdrawn every hour and the Luciferase activity was measured. The relative fold change to the unstimulated control was calculated and assigned. (D) qRT-PCR analysis of *id1* and *smad6.* The cells were stimulated with the indicated concentrations for 15 minutes and Dorsomorphin was given to the cells. Every hour one sample was lysed and frozen at −80°C until the further processing.

A recent study revealed that the duration of stimulation is critical for the cellular response [21]. To better understand the signaling dynamics and the critical parameters, we analyzed how cells respond to a short time receptor stimulus of only 15 minutes. We measured Luciferase activity over 30 h as described above. Instead of keeping the stimulation medium on the cells until the end of the experiment we, however, replaced it with basal DMEM medium after 15 minutes. The relative Luciferase activity of 0.1 nM, 1 nM and 10 nM BMP2 in case of the wash-away treatment displayed an activity pattern similar to that seen during continuous treatment (Fig. 3A, Figure S15A and B). Hallmarks of the measured responses were concentration- and time-dependency as well as the oscillating progression with significant activity increases and decreases. Certainly, relative Luciferase activities (fold changes) were lower for 15 minutes stimulation than for continuous treatment.

As before, we verified the reporter gene expression data by qRT-PCR analysis (Fig. 3B) of endogenous target genes. 15 minutes post receptor activation both target genes showed increased expression and sustained it over 8 h, again displaying oscillations for both 0.1 nM and 1 nM BMP2. This fact becomes even more obvious when looking at the single experiment data (Figure S16).

### Short-time Smad-phosphorylation leads to decreased and downregulated gene expression

Short-time receptor stimulation was sufficient to activate target gene expression for as long as continuous stimulation. To examine the influence of the receptor kinase on this mechanism, we next wanted to examine how the gene expression profile changes when the receptor kinase is inhibited and no further Smad-proteins can be activated. We therefore stimulated the cells with BMP2 as before, but administered Dorsomorphin (BMP receptor type I kinase inhibitor) to the cells 15 minutes later (Fig. 3C, Figure S17A and B). Compared to the other treatments, the Luciferase assays again show an oscillatory curve shape, but decreased activity fold changes as well as a complete termination after 12 h stimulation time.

The qRT-PCR experiments confirmed this observation. *id1* was upregulated after 1 h stimulation-time with 1 nM BMP2, but later downregulated to a level below the basal level (Fig. 3D). Cells that were stimulated with 0.1 nM BMP and the non-stimulated control cells showed an immediate downregulation of *id1* after 1 h. In the case of *smad6,* Dorsomorphin treatment resulted in an immediate downregulation for all tested ligand concentrations. These data show that the half-life time of the receptor-kinase activity is 0.5 h.

### Different pathway components contribute to the oscillatory response

**Figure 4:**
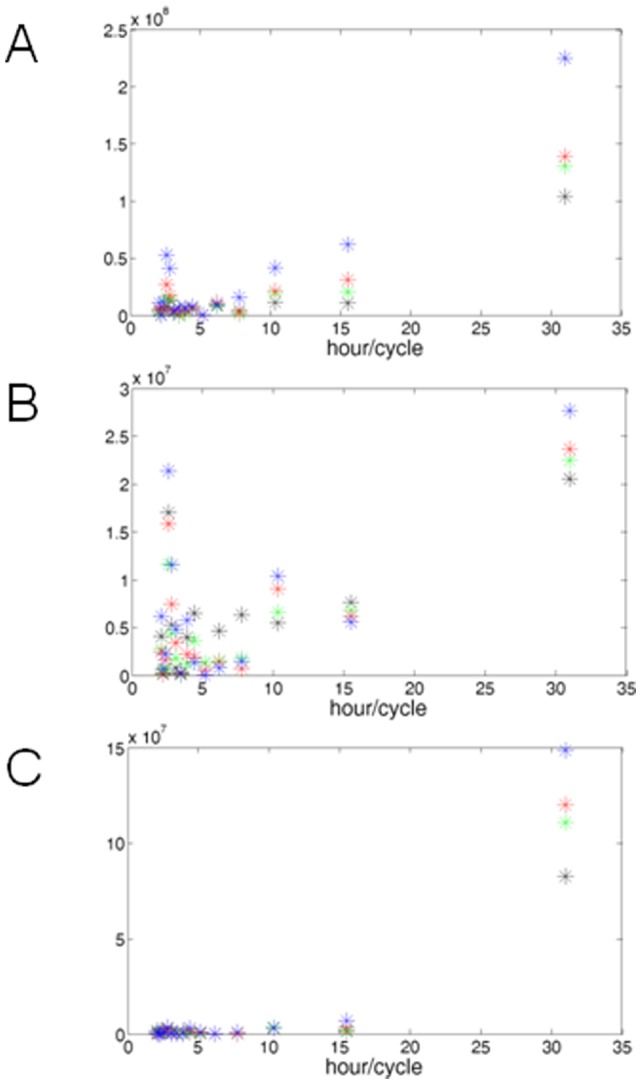
Fast Fourier Transformation (FFT) of the Luciferase data. The absolute values of one Gaussia Luciferase experiment triplet were entered to the MATLAB software and transformed using the “fft” comand. The blue crosses illustrate the stimulation with 10 nM BMP, the red crosses the stimulation with 1 nM BMP, the green crosses with 0.1 nM BMP and the black crosses show the non-stimulated control. (A) FFT of the continuous stimulation experiment. (B) FFT of the short-time receptor stimulus. (C) FFT of the short-time Smad-phosphorylation.

The gene expression experiments revealed that continuous as well as short-term receptor stimulation result in sustainable and oscillating cellular responses, whereas short-term Smad activation leads to abbreviated and decreased responses. We next studied the results of the Luciferase experiments with mathematical methods to compare components of the detected oscillation patterns of the three treatments. Figure 4 shows Fast Fourier Transformations (FFT) of the absolute Luciferase activities of the different cell treatments. Interestingly, the plots of the continuous and the short-term receptor stimulus treatments show the same prominent oscillation components (Fig. 4A and B). Both show one slow oscillation on a 31 h frequency which appears to be concentration dependent, as the amplitudes rise with higher ligand concentrations. Moreover, at about 16 h/cycle there is an oscillation that increases in proportion to the ligand amount in the case of the continuous stimulation, but it is inversely proportional to the short-time treatment. Two more at about 10.5 and 2.8 h/cycle are proportional to the BMP2 concentration in both cases. Finally, another interesting oscillation was found at 2.5 h/cycle, which is proportional to the ligand concentration for the continuous stimulation and for the short receptor stimulus treatment. In contrast to that is the treatment with the receptor kinase inhibitor (Fig. 4C). Here only one distinctive oscillation at 31 h/cycle was identified, suggesting that different components of the signaling pathway contribute to the overall oscillatory behaviour.

## Discussion

In recent years, the analysis of signaling modulation and networking responsible for the regulation of target gene expression has gained increasing attention. A great deal of work has been done on modeling a series of different signaling pathways including the TGF-ß signaling pathway [21], [26]–[28]. Since BMP and TGF-ß signaling share the co-Smad, the inhibitory Smads as well as one receptor subtype, one could assume that the models of the TGF-ß pathway are conferrable to the BMP-pathway. In this study we examined the dynamics of the BMP signaling pathway and could show profound differences to the TGF-ß signaling dynamics.

For the TFGß-Smad2/3 pathway, it is well known that Smad2/3 strongly accumulate in the nucleus upon stimulation with very low TGF-ß concentrations in the picomolar range [20], [29]. Our data indicate that this cannot be attributed to Smad1 in c2c12 cells. Instead we found that only the phospho-Smad1 level in the nuclei increases in proportion to the ligand-concentration. A similar effect has been described for Smad1 in a B-cell lymphoma cell line after stimulation with TGF-ß [30]. Furthermore, studies in *Xenopus* embryos also suggest that the pathway activity has little effect on the localization of Smad1 [31]. Reasons for this difference within the Smad family are unknown, yet two studies indicate that differences in nuclear export signals might play a role [32], [33]. Our results strongly suggest a constant, basal nucleocytoplasmic shuttling of Smad1, which is not significantly modulated by stimulation up to 1 nM BMP2. However, it has been shown that stimulation with 10 nM BMP2 leads to a clear nuclear accumulation of Smad1 [34]. As a conclusion, Smad1-shuttling dynamics are dose-dependent with a threshold-concentration in the range between 1 nM and 10 nM. The Smad1 import-rate exceeds the export-rate and the proteins accumulate in the nucleus. Reasons for that might be lacking dephosphorylation, more phospho-Smad1 proteins per complex meaning longer resting time on DNA or that export-proteins have poorer binding efficiency for phospho-Smad1 proteins; many possibilities for this phenomenon, but the real cause remains unexplored.

Concerning the TGF-ß/Smad pathway, it is known that upon sustained stimulation the amount of phospho-Smad2 increases to a maximum and then decreases to a constant level over 8 h [21]. This fact points to a correlating gene expression profile. Our data also show a maximum of *smad6* expression after 2 h with subsequent relative peaks at two-hourly intervals. We conclude that there is a basal oscillation mechanism of the BMP-induced expression of *id1* and *smad6.* Oscillating target gene transcription is supported by the fact that *hes1* expression, which is controlled by BMP2 in embryonic stem cells, has been found to oscillate and a *hes1*-promoter driven Luciferase mimics this oscillatory expression [35]. We further conclude that the oscillatory frequency is not influenced by stimulation intensity (BMP concentration). Instead, the amplitudes are clearly dose-dependent. The discriminated signaling response in the case of Dorsomorphin treatment might be attributed to the input strength in combination with the inhibitor potency. Shortly after continuous stimulation with 1 nM BMP2 nuclear phospho-Smad1 level is considerably higher than the level with 0.1 nM. Thus after stimulation with BMP2 and subsequent kinase-inhibitor treatment, the phospho-Smad1 level is sufficient to exclusively trigger expression of very early target genes. Since phospho-Smad1 proteins are rapidly degraded by E3 ubiquitin ligases [36] expression of later target genes expires. Mouse models for lung cancer associate elevated BMP2 levels with increased malignancy [37], promoted lung tumor growth [38] and stimulated angiogenesis in developing tumors [39]. Our study shows, that the BMP target gene transcription is dose-dependent. As BMP2 has been found to induce cell cycle regulators [40], this might be a reason for pathological proliferation. Consequently, disease conditions might trigger Smad1 accumulation and thus break physiological regulatory pathway mechanisms.

Comparison of continuous exposure with kinase-inhibitory and wash-away treatment demonstrated that target gene oscillation is directly dependent on the receptor kinase. Furthermore, once ligand-bound, thus activated, receptor complexes keep up target-gene stimulation for at least 8 h and in wave-like forms without receptor reactivation. It is known that Smad6 is an inhibitor of BMP signaling and continuous expression of Smad6 leads to the inhibition of phospho-Smad1 formation [41]. As our results show that *smad6* oscillates with 1 h delay to *id1*, Smad6 is a negative feedback regulator and may be an important regulator for the wave-like response. Possible advantages of oscillating responses are minimized exposure to high levels of ligand. Furthermore, transforming stimuli into oscillatory signals is more robust against noise in the input signal and signal propagation [42]. However, the real causes for these oscillations remain unclear.

During embryogenesis, body patterning and axis formation are determined by morphogen gradients and different cell fates result from distinct gene expression profiles. Furthermore, BMPs are used as therapeutics in the clinic. Our data on signal half-life, the influence of BMP2 concentration, exposure time and receptor inhibition on the temporal course of target-gene-expression create a basis for a novel mathematical model for the BMP signaling pathway that could be used to improve drug compositions, amounts and administration for the compensation of developmental defects and the patients benefit.

## Materials and Methods

### Cell Culture

The mouse myoblast cell line c2c12 (purchased from http://lgcstandards-atcc.org/. Accessed 2013 March 3.) was cultured in DMEM supplemented with 10% FCS (PAA) and penicillin/streptomycin at 37°C in a humidified atmosphere. To examine the cellular response of the cells to BMP2 stimulation, cells were starved over night in basal DMEM. Transfections were performed using the Fugene HD transfection reagent (Roche) following the manufacturer's instructions.

### Generation of reporter construct and cell line

The BRE-Luciferase reporter construct contains a dimer of a published BMP responsive element [43] in front of a MLP minimal promoter and the Gaussia Luciferase gene; furthermore it comprises an independent mCherry-Zeocin fusion under control of a CMV-promoter. The genes are flanked by a tol2 recognition site for generation of a stable cell line including both reporters.

For generation of the c2c12-BRE_Luc cell line, the tol2 transposase system [44] was used. Cells were cotransfected with a construct containing the coding sequence of the tol2 transposase under control of the CMV-promoter and the BRE_Luc construct at a ratio of 2∶1, and subsequently selected with Zeocin for two weeks. Single colonies were picked, expanded and then checked for correct function of the BRE_Luc construct.

### Luciferase Assay

The stable c2c12-BRE_Luc cells were seeded out at a density of 15000cells/cm^2^ in 6 well plates in the morning in complete medium and starved over night. On the next day, 50 µl medium of all wells was withdrawn hourly for 4 hours to generate the baseline. After that cells were stimulated with 0 nM, 0.1 nM, 1 nM or 10 nM BMP2 (kind gift of Walter Sebald, Würzburg) in starvation medium. Three different cell treatments were performed: (1) cells were permanently stimulated with BMP2 (continuous stimulation), (2) cells were stimulated for 15 minutes, then the pathway was inhibited by the administration of 10 mM Dorsomorphin (Sigma-Aldrich) or (3) cells were stimulated for 15 minutes, then the stimulation medium was removed and fresh starvation medium was given to the cells. Then every hour 50 µl of the culture medium was removed and stored at 4°C untill measurement. An equal volume of fresh starvation medium was administrated to the cells, to keep a constant medium volume over the whole time of the experiment. After 30 h stimulation time, the Luciferase activity was measured with the Promega GloMax 96 Microplate Luminometer and a final concentration of 20 µM Coelenterazine (Synchem OHG) for the enzyme reaction. Data were evaluated using Student's t-test.

### Immunofluorescent stainings

c2c12 wildtype cells were seeded out on glass coverslips and starved over night. Then cells were stimulated with 0 nM, 0.1 nM or 1 nM BMP2 for indicated time points. Immunofluorescent staining was performed as previously described [45] using anti-Smad1 antibody (Santa Cruz, sc-9765**),** Alexa Fluor 488 secondary antibody (Invitrogen), Hoechst 33258 (Molecular probes) for DNA staining and CellMask Orange (Invitrogen) for cell membrane staining. Confocal stacks were taken at room temperature using a Nikon Eclipse Ti confocal microscope and data were processed using Volocity 3D Image Analysis Software (Improvision).

### Live Smad1 shuttling

A meGFP-Smad1 fusion protein was cloned in front of a CMV-promoter. This vector was cotransfected with a H2B-mCherry fusion construct in a 2∶1 ratio into c2c12 wildtype cells and starved over night. On the next day, cells were treated with 0 nM BMP2, 0 nM BMP2 with 10 ng/ml Leptomycin B, 1 nM BMP2 or 1 nM BMP2 with 10 ng/ml Leptomycin B (Sigma-Aldrich) in starvation medium. Then the cells were incubated at 37°C and imaged for 1 h with the Nikon Eclipse Ti confocal microscope. The resulting data were processed using Volocity 3D Image Analysis Software (Improvision).

### Real-Time PCR

Total RNA was isolated from cell cultures using the Total RNA Isolation Reagent (AB Gene). Samples were digested with DNAseI (Fermentas) to exclude gDNA contamination followed by cDNA synthesis (Fermentas). Real-time PCR was performed on 25 ng cDNA using SYBR Green reagent and the following primer pairs in single reactions: EF1a forward 5′- TCAGGAGGAGACCACACCTT-3′ and reverse 5′- ATATCCACAGGCAGCAAACA-3′; ID1 forward 5′- AGAACCGCAAAGTGAGCAAG-3′ and reverse 5′- GTGGTCCCGACTTCAGACTC-3′; Smad6 forward 5′- CAAGATCGGTTTTGGCATACTG -3′ and reverse 5′- GTCGGGGAGTTGACGAAGAT -3′. PCRs were run at the following conditions: 5 minutes denaturation at 95°C; 40 amplification cycles, each including denaturation at 95°C for 15 seconds, annealing for 20 seconds at 55°C and 20 seconds extension at 72°C. The products of the primers pairs were of approximately the same size and had similar melting points, enabeling direct comparison of the transcripts. The results are averages from four independent experiments. Data were evaluated using Student's t-test.

### Mathematical Analysis

The results from the Luciferase experiments were entered to the MATLAB software and transformed using the fft algorithm.

## Supporting Information

Figure S1 **Nucleocytoplasmic shuttling of Smad1.** c2c12 wt cells were seeded out on glass coverslips and starved over night. On the next day, the cells were stimulated with 0 nM, 0.1 nM or 1 nM BMP2 for 0 min, 30 min, 1 h, 1.5 h or 2 h. Then the cells were fixed, sampled with anti-Smad1 primary antibody and Alexa 488-secondary antibody and confocal images were acquired.
(TIF)

Figure S2 **Real-color images according to the pseudocolor images of** **Fig. 1B**. These pictures show the real green fluorescence of the Smad1-GFP fusion protein.
(TIF)

Figure S3 **Nuclear pSmad/total Smad1 ratio after stimulation with BMP2.** c2c12 wt cells were seeded out on coverslips and starved over night. On the next day, the cells were stimulated with (A) 0 nM, (B) 0.1 nM or (C) 1 nM BMP2 for 0 min, 20 min, 40 min or 60 min. After fixation with PFA and methanol-permeabilization, the cells were sampled with a primary phospho-Smad1/5/8 and secondary Alexa594-secondary antibody. Further immunostaining was performed using anti-Smad1 primary antibody and Alexa 488-secondary antibody and a following incubation with Hoechst was executed for nuclear staining. Then confocal stacks were taken and processed using Volocity 3D software. (D)The fluorescence intensities of the two Alexa-antibodies were taken to calculate the phospho-Smad/Smad ratio. The bar graphs in the lower panel describe significant increases/decreases between two treatment groups for the respective time points. For example, is the nuclear pSmad/Smad ratio after 40 min stimulation time with 1 nM BMP significantly increased compared to the 40 min non-stimulated situation.
(TIF)

Figure S4 **Phase contrast images of c2c12 cells.** The pictures show the typical variable morphology of the c2c12 cell line.
(TIF)

Figure S5 **Progression of the absolute Gaussia Luciferase activity upon sustained stimulation with BMP2.** c2c12_BRE-Luc cells was seeded out in 6-well plates and starved over night. On the next day, cells were stimulated with 0 nM, 0.1 nM, 1 nM or 10 nM BMP2. 50 µl medium from every well were removed hourly and stored at 4°C. All samples were measured on the same day with the same Coelenterazine-solution. The assigned values represent averages from independent triplets out of one experiment.
(TIF)

Figure S6 **Decrease of Gaussia Luciferase activity after incubation at 37°C for 1 h.** c2c12_BRE-Luc cells were seeded out and starved over night. On the next day they were stimulated with 0 nM or 10 nM BMP2. After 12 h stimulation time, 50 µl from every well were removed twice every hour. One sample was stored at 4°C until the measurement and the other sample was incubated for one additional hour at 37°C and then stored at 4°C until the measurement. All samples were measured on the same day with the same Coelenterazine-solution. All values represent averages from independently measured triplets of the same experiment.
(TIF)

Figure S7 **Progression of absolute Gaussia Luciferase activity upon sustained stimulation with TNFα.** c2c12 cells were seeded out in 6-well plates, transiently transfected with a NFkb-binding site Luciferase reporter and starved over night. On the next day, cells were stimulated with 0 ng/ml or 10 ng/ml TNFα. 50 µl medium from every well were removed hourly and stored at 4°C. All samples were measured on the same day with the same Coelenterazine-solution.
(TIF)

Figure S8 **Two further independent 30** **h Luciferase experiments (A) and (B) upon sustained stimulation with BMP2.**
(TIF)

Figure S9 **Curve progressions of the four independent continuously stimulated qRT-PCR experiments of (A)** ***id1*** **after stimulation with 1** **nM BMP2, (B)** ***id1*** **after stimulation with 0.1** **nM BMP2, (C)** ***smad6*** **after 1** **nM BMP2 and (D)** ***smad6*** **after 0.1** **nM BMP2.**
(TIF)

Figure S10 **Higher temporal resolution of the target gene curve progression.** c2c12_BRE-Luc cells were seeded out and starved over night. Then the cells were stimulated with 0 nM, 0.1 nM or 1 nM BMP2 and harvested at the indicated time points after stimulation. The qRT-PCR anaylsis of the BMP target genes *id1* and *smad6* as well as the housekeeping gene *ef1a* followed. The relative fold change to the housekeeping gene was calculated and depicted. This figure represents the average of two independent experiments.
(TIF)

Figure S11 **Higher temporal resolution of the target gene curve progression.** c2c12_BRE-Luc cells were seeded out and starved over night. Then the cells were stimulated with 0 nM or 1 nM BMP2 and harvested at the indicated time points after stimulation. The qRT-PCR anaylsis of the BMP target genes *id1* and *smad6* as well as the housekeeping gene *ef1a* followed. The relative fold change to the housekeeping gene was calculated and depicted. This figure represents the average of two independent experiments.
(TIF)

Figure S12 **Analysis of BMP target gene transcription after long time stimulation.** c2c12_BRE-Luc cells were seeded out and starved over night. Then the cells were stimulated with 0 nM, 0.1 nM or 1 nM BMP2 and harvested at the indicated time points after stimulation. The qRT-PCR anaylsis of the BMP target genes *id1* and *smad6* as well as the housekeeping gene *ef1a* followed. The relative fold change to the housekeeping gene was calculated and depicted. This figure represents the average of two independent experiments.
(TIF)

Figure S13 **Raw data from two single qRT-PCR experiments.** (A) Triplet of ct-values from one experiment analyzing *ef1a* and *id1* after stimulation with 1 nM BMP2. (B) Triplet of ct-values from one experiment analyzing *ef1a* and *smad6* after stimulation with 0.1 nM BMP2.
(TIF)

Figure S14 **Quantitative real-time PCR was performed on the Gaussia Luciferase gene of the stably transgenic cell line.** The cells were stimulated with 0 nM (black) or 1 nM (red) BMP2 and every hour one sample was lysed and frozen at −80°C until the further processing.
(TIF)

Figure S15 **Two further independent 30 h Luciferase experiments (A) and (B) upon removal of the stimulation medium.**
(TIF)

Figure S16 Curve progressions of the four independent short-time stimulation qRT-PCR experiments of (A) *id1* after stimulation with 1 nM BMP2, (B) *id1* after stimulation with 0.1 nM BMP2, (C) *smad6* after 1 nM BMP2 and (D) *smad6* after 0.1 nM BMP2.
(TIF)

Figure S17 **Two further independent 30** **h Luciferase experiments (A) and (B) with additional Dorsomorphin treatment.**
(TIF)
