# Supplementary figures and images for: Bursted BMP Triggered Receptor Kinase Activity Drives Smad1 Mediated Long-Term Target Gene Oscillation in c2c12 Cells
Source: PLoS One. 2013 Apr 1;8(4):e59442. doi: 10.1371/journal.pone.0059442 (PMC3613406; doi:10.1371/journal.pone.0059442)

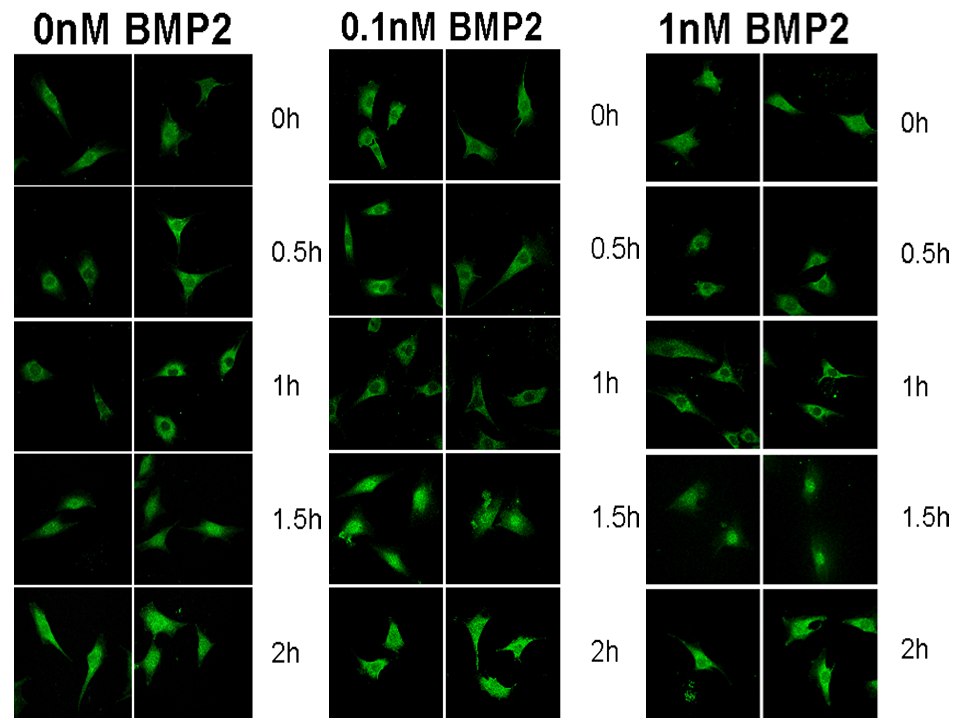

Supplement: Figure S1 — Nucleocytoplasmic shuttling of Smad1. c2c12 wt cells were seeded out on glass coverslips and starved over night. On the next day, the cells were stimulated with 0 nM, 0.1 nM or 1 nM BMP2 for 0 min, 30 min, 1 h, 1.5 h or 2 h. Then the cells were fixed, sampled with anti-Smad1 primary antibody and Alexa 488-secondary antibody and confocal images were acquired. (TIF) [file pone.0059442.s001.tif]

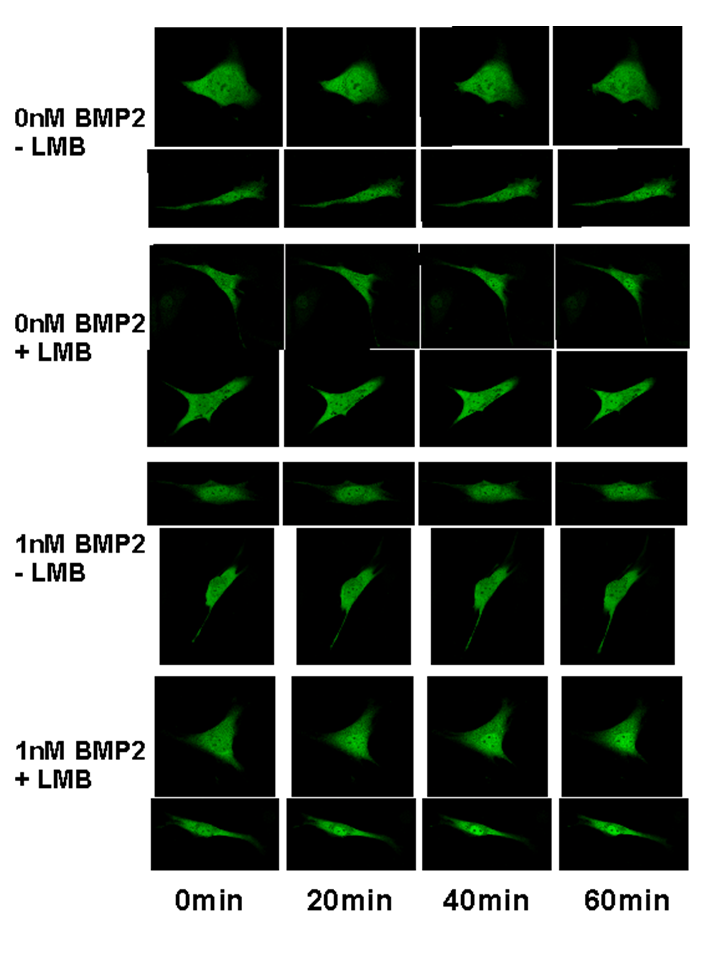

Supplement: Figure S2 — Real-color images according to the pseudocolor images of Fig. 1B . These pictures show the real green fluorescence of the Smad1-GFP fusion protein. (TIF) [file pone.0059442.s002.tif]

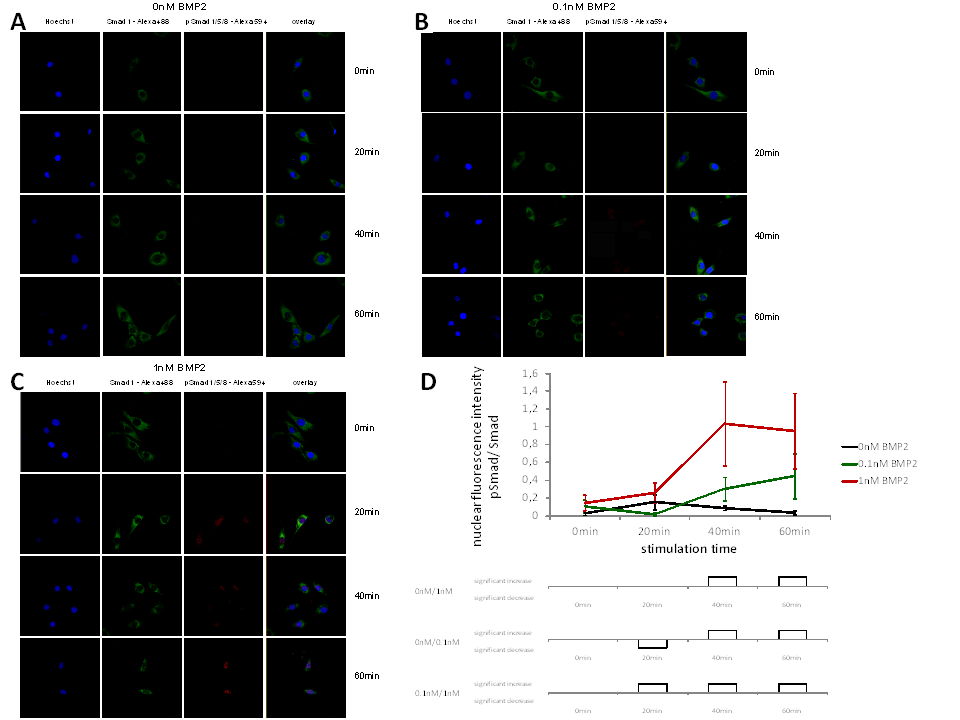

Supplement: Figure S3 — Nuclear pSmad/total Smad1 ratio after stimulation with BMP2. c2c12 wt cells were seeded out on coverslips and starved over night. On the next day, the cells were stimulated with (A) 0 nM, (B) 0.1 nM or (C) 1 nM BMP2 for 0 min, 20 min, 40 min or 60 min. After fixation with PFA and methanol-permeabilization, the cells were sampled with a primary phospho-Smad1/5/8 and secondary Alexa594-secondary antibody. Further immunostaining was performed using anti-Smad1 primary antibody and Alexa 488-secondary antibody and a following incubation with Hoechst was executed for nuclear staining. Then confocal stacks were taken and processed using Volocity 3D software. (D)The fluorescence intensities of the two Alexa-antibodies were taken to calculate the phospho-Smad/Smad ratio. The bar graphs in the lower panel describe significant increases/decreases between two treatment groups for the respective time points. For example, is the nuclear pSmad/Smad ratio after 40 min stimulation time with 1 nM BMP significantly increased compared to the 40 min non-stimulated situation. (TIF) [file pone.0059442.s003.tif]

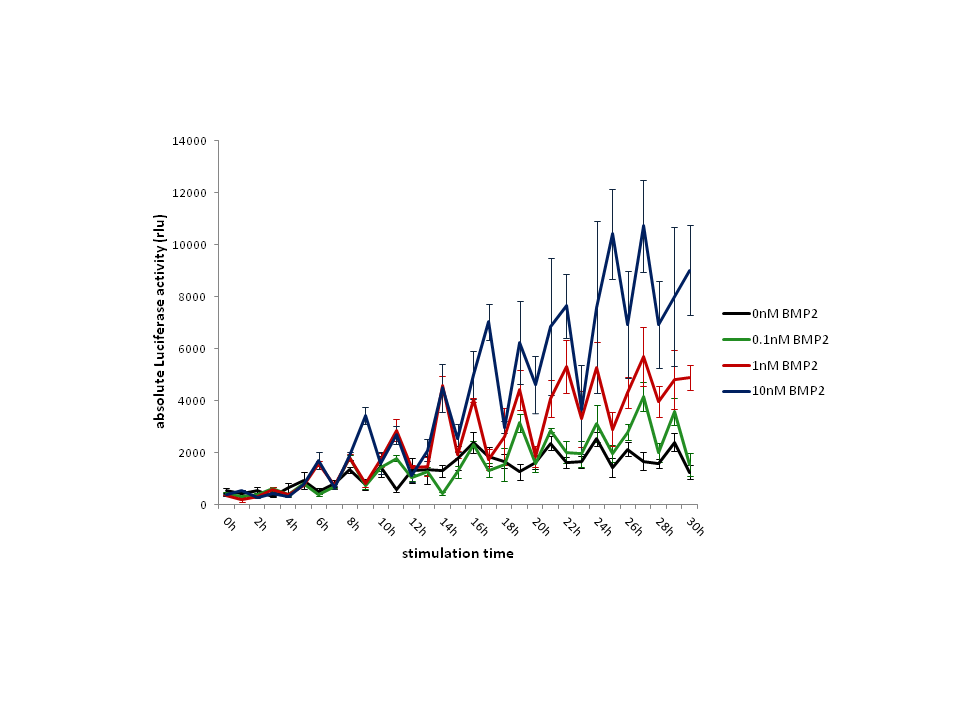

Supplement: Figure S5 — Progression of the absolute Gaussia Luciferase activity upon sustained stimulation with BMP2. c2c12_BRE-Luc cells was seeded out in 6-well plates and starved over night. On the next day, cells were stimulated with 0 nM, 0.1 nM, 1 nM or 10 nM BMP2. 50 µl medium from every well were removed hourly and stored at 4°C. All samples were measured on the same day with the same Coelenterazine-solution. The assigned values represent averages from independent triplets out of one experiment. (TIF) [file pone.0059442.s005.tif]

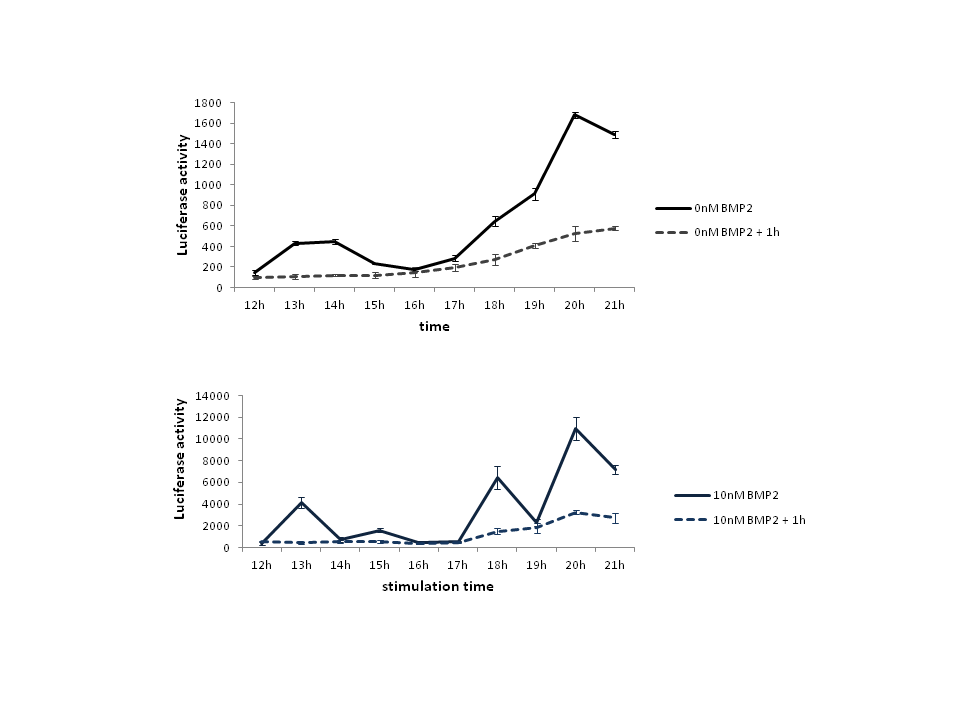

Supplement: Figure S6 — Decrease of Gaussia Luciferase activity after incubation at 37°C for 1 h. c2c12_BRE-Luc cells were seeded out and starved over night. On the next day they were stimulated with 0 nM or 10 nM BMP2. After 12 h stimulation time, 50 µl from every well were removed twice every hour. One sample was stored at 4°C until the measurement and the other sample was incubated for one additional hour at 37°C and then stored at 4°C until the measurement. All samples were measured on the same day with the same Coelenterazine-solution. All values represent averages from independently measured triplets of the same experiment. (TIF) [file pone.0059442.s006.tif]

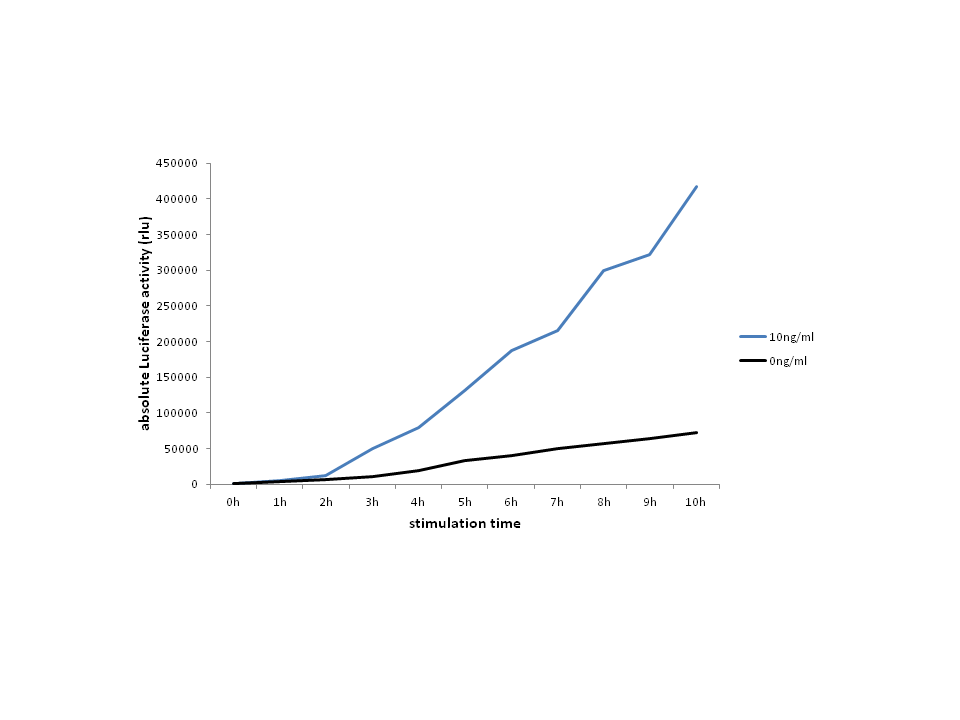

Supplement: Figure S7 — Progression of absolute Gaussia Luciferase activity upon sustained stimulation with TNFα. c2c12 cells were seeded out in 6-well plates, transiently transfected with a NFkb-binding site Luciferase reporter and starved over night. On the next day, cells were stimulated with 0 ng/ml or 10 ng/ml TNFα. 50 µl medium from every well were removed hourly and stored at 4°C. All samples were measured on the same day with the same Coelenterazine-solution. (TIF) [file pone.0059442.s007.tif]

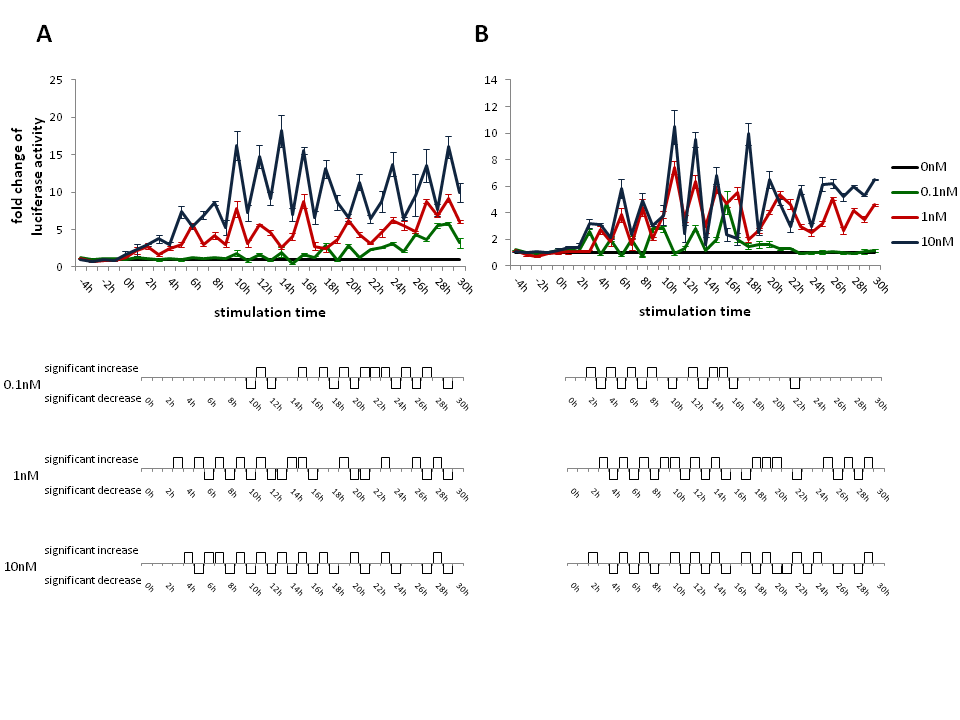

Supplement: Figure S8 — Two further independent 30 h Luciferase experiments (A) and (B) upon sustained stimulation with BMP2. (TIF) [file pone.0059442.s008.tif]

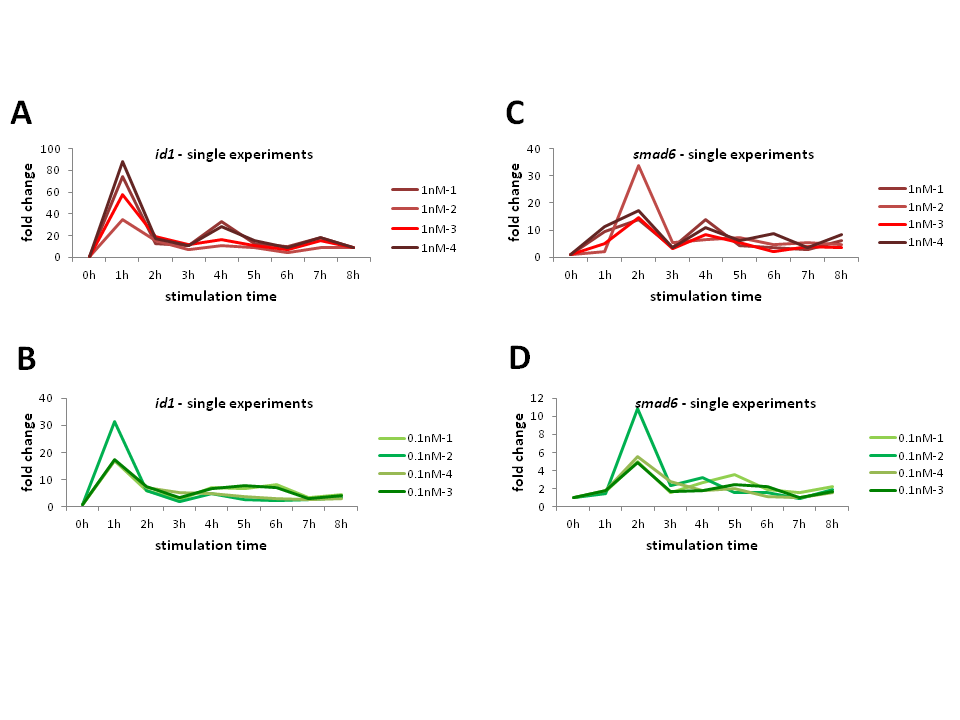

Supplement: Figure S9 — Curve progressions of the four independent continuously stimulated qRT-PCR experiments of (A) id1 after stimulation with 1 nM BMP2, (B) id1 after stimulation with 0.1 nM BMP2, (C) smad6 after 1 nM BMP2 and (D) smad6 after 0.1 nM BMP2. (TIF) [file pone.0059442.s009.tif]

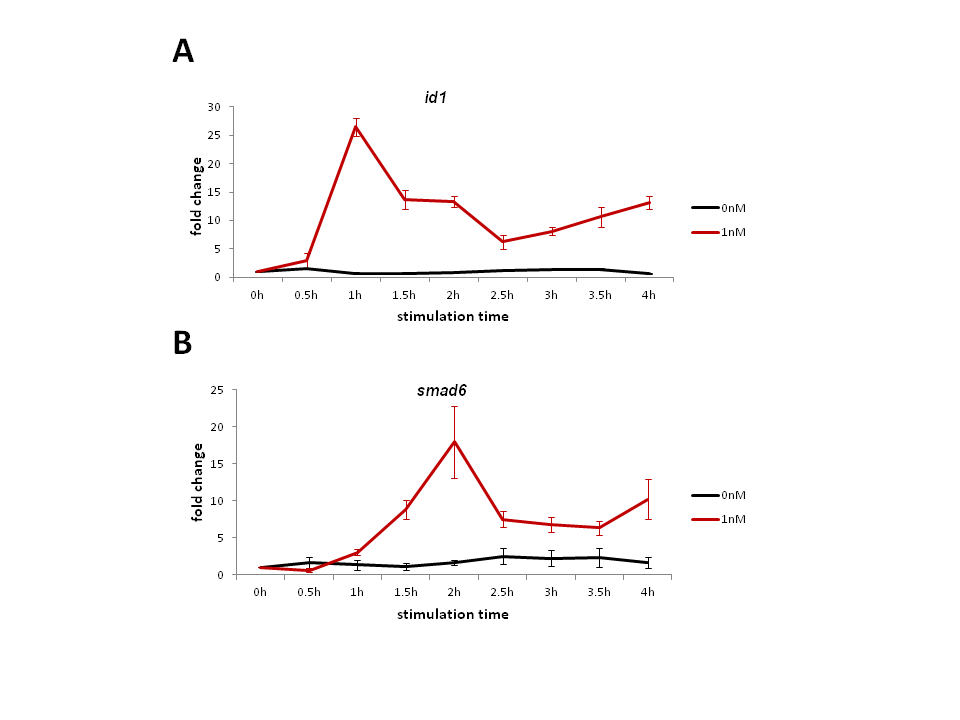

Supplement: Figure S10 — Higher temporal resolution of the target gene curve progression. c2c12_BRE-Luc cells were seeded out and starved over night. Then the cells were stimulated with 0 nM, 0.1 nM or 1 nM BMP2 and harvested at the indicated time points after stimulation. The qRT-PCR anaylsis of the BMP target genes id1 and smad6 as well as the housekeeping gene ef1a followed. The relative fold change to the housekeeping gene was calculated and depicted. This figure represents the average of two independent experiments. (TIF) [file pone.0059442.s010.tif]

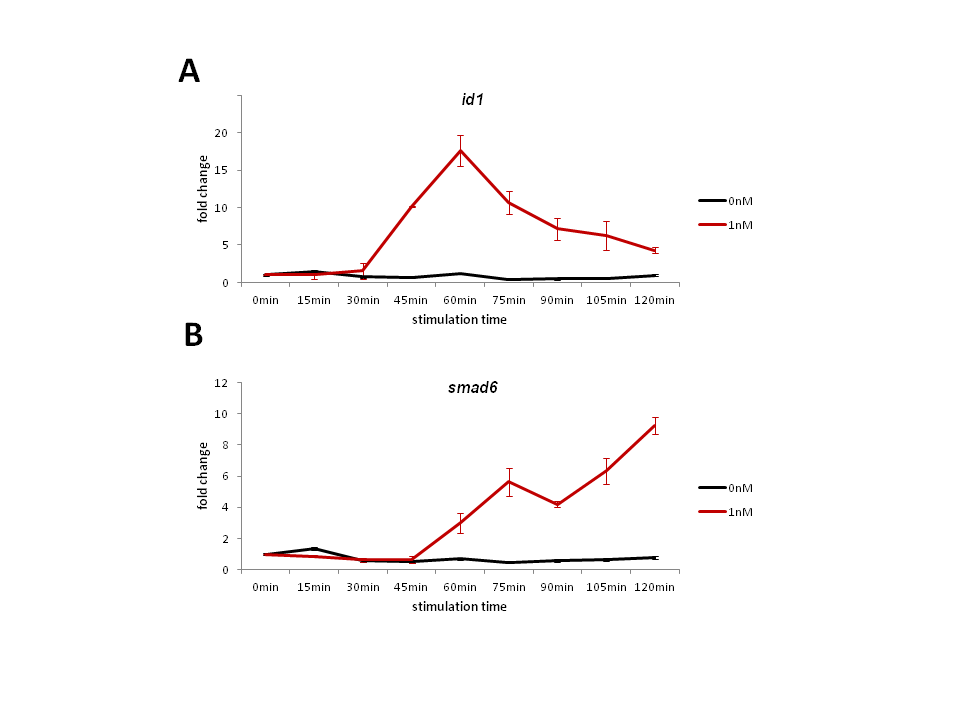

Supplement: Figure S11 — Higher temporal resolution of the target gene curve progression. c2c12_BRE-Luc cells were seeded out and starved over night. Then the cells were stimulated with 0 nM or 1 nM BMP2 and harvested at the indicated time points after stimulation. The qRT-PCR anaylsis of the BMP target genes id1 and smad6 as well as the housekeeping gene ef1a followed. The relative fold change to the housekeeping gene was calculated and depicted. This figure represents the average of two independent experiments. (TIF) [file pone.0059442.s011.tif]

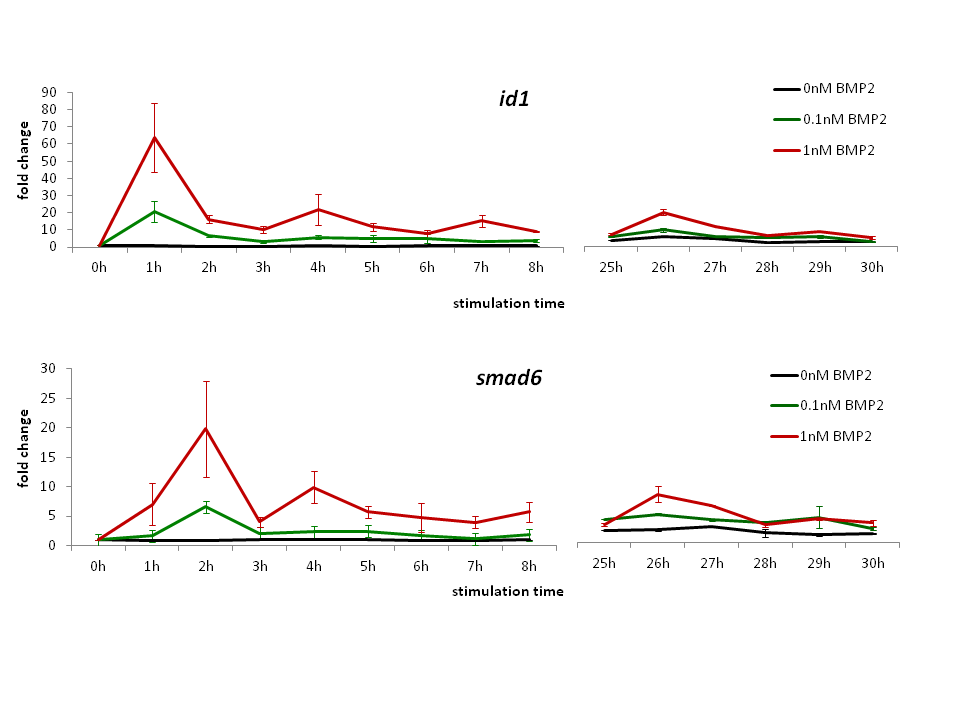

Supplement: Figure S12 — Analysis of BMP target gene transcription after long time stimulation. c2c12_BRE-Luc cells were seeded out and starved over night. Then the cells were stimulated with 0 nM, 0.1 nM or 1 nM BMP2 and harvested at the indicated time points after stimulation. The qRT-PCR anaylsis of the BMP target genes id1 and smad6 as well as the housekeeping gene ef1a followed. The relative fold change to the housekeeping gene was calculated and depicted. This figure represents the average of two independent experiments. (TIF) [file pone.0059442.s012.tif]

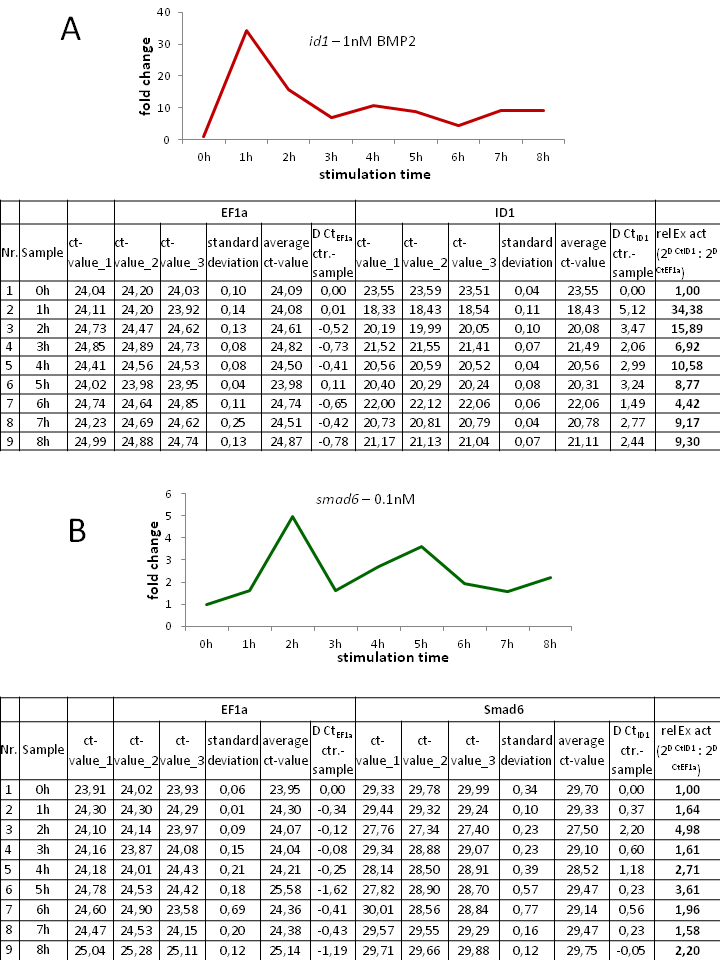

Supplement: Figure S13 — Raw data from two single qRT-PCR experiments. (A) Triplet of ct-values from one experiment analyzing ef1a and id1 after stimulation with 1 nM BMP2. (B) Triplet of ct-values from one experiment analyzing ef1a and smad6 after stimulation with 0.1 nM BMP2. (TIF) [file pone.0059442.s013.tif]

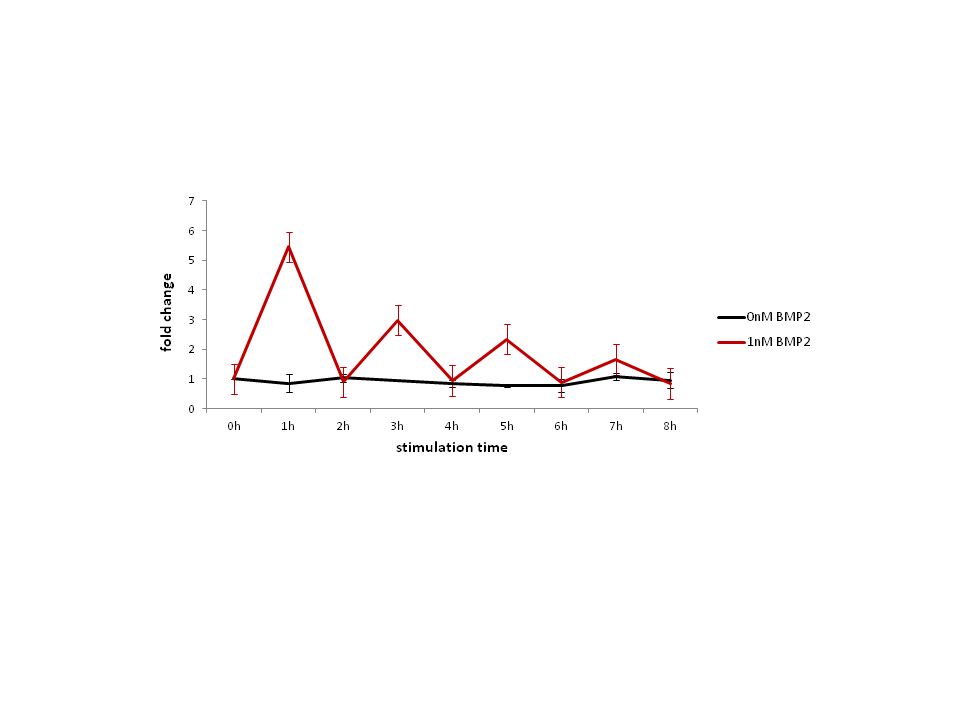

Supplement: Figure S14 — Quantitative real-time PCR was performed on the Gaussia Luciferase gene of the stably transgenic cell line. The cells were stimulated with 0 nM (black) or 1 nM (red) BMP2 and every hour one sample was lysed and frozen at −80°C until the further processing. (TIF) [file pone.0059442.s014.tif]

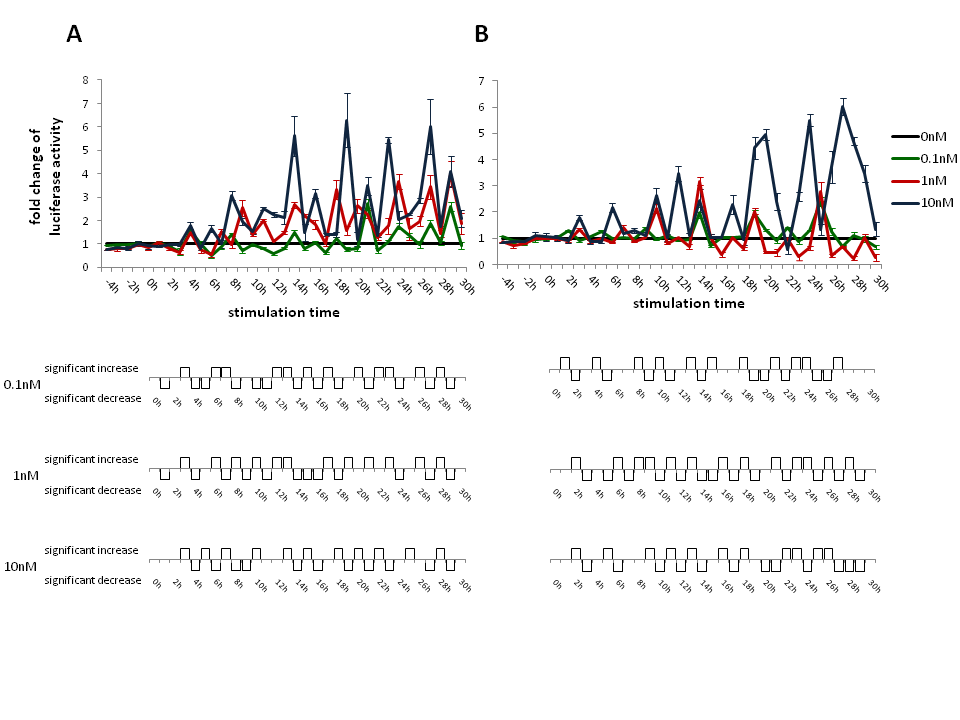

Supplement: Figure S15 — Two further independent 30 h Luciferase experiments (A) and (B) upon removal of the stimulation medium. (TIF) [file pone.0059442.s015.tif]

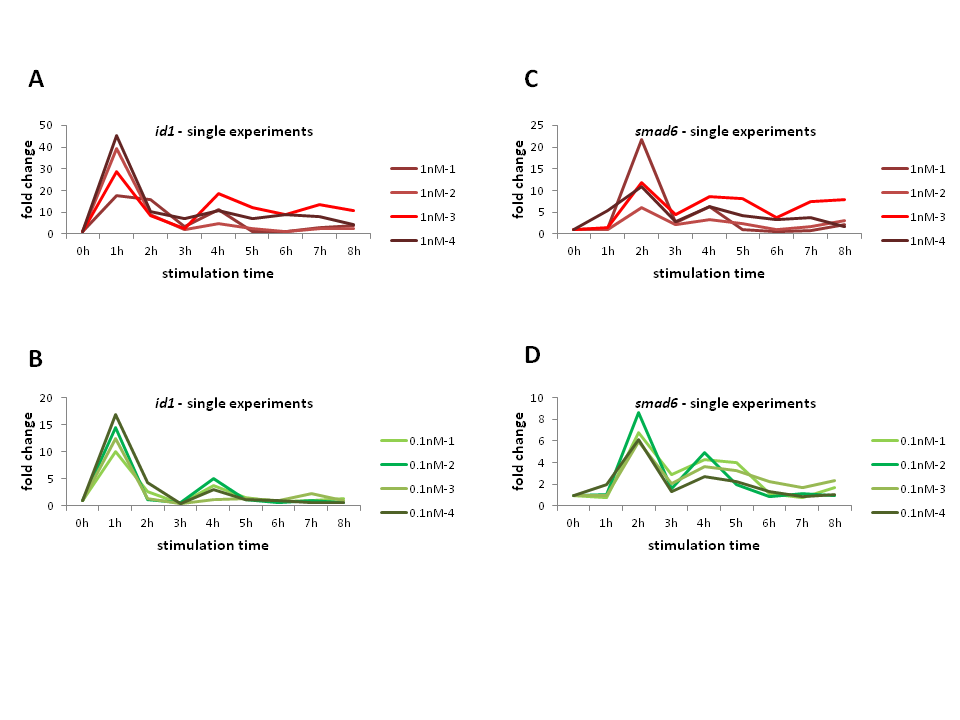

Supplement: Figure S16 — Curve progressions of the four independent short-time stimulation qRT-PCR experiments of (A) id1 after stimulation with 1 nM BMP2, (B) id1 after stimulation with 0.1 nM BMP2, (C) smad6 after 1 nM BMP2 and (D) smad6 after 0.1 nM BMP2. (TIF) [file pone.0059442.s016.tif]

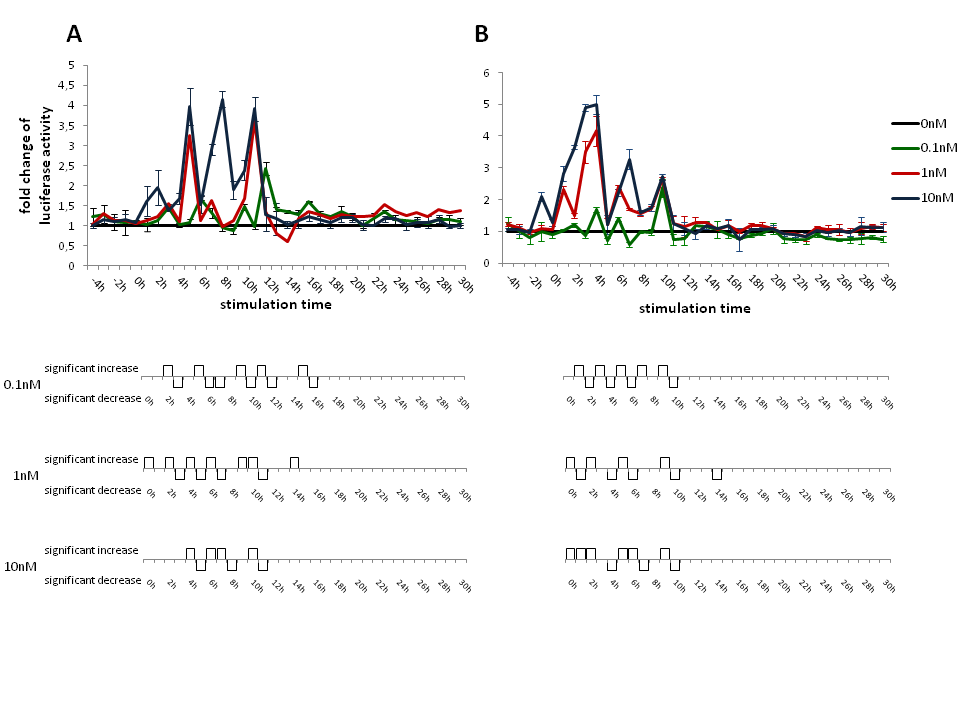

Supplement: Figure S17 — Two further independent 30 h Luciferase experiments (A) and (B) with additional Dorsomorphin treatment. (TIF) [file pone.0059442.s017.tif]
